# Uremic Pruritus in Hemodialysis: Mechanisms, Burden, and Emerging Therapies

**DOI:** 10.3390/jcm15020494

**Published:** 2026-01-08

**Authors:** Marina Kljajić, Ena Parać, Armin Atić, Nikolina Bašić-Jukić

**Affiliations:** 1 Department of Internal Medicine, University Hospital Center Zagreb, 10000 Zagreb, Croatianbasic@kbc-zagreb.hr (N.B.-J.); 2 School of Medicine, University of Zagreb, 10000 Zagreb, Croatia; 3 Children’s Hospital Zagreb, 10000 Zagreb, Croatia

**Keywords:** uremic pruritus, chronic kidney disease, hemodialysis

## Abstract

**Background/Objectives:** Uremic pruritus is a common complication in patients with end-stage kidney disease undergoing maintenance hemodialysis. Despite its high prevalence and substantial impact on sleep, psychological well-being, and overall quality of life, its pathophysiology remains multifactorial and incompletely understood. This narrative review summarizes contemporary evidence (2015–2025) on therapeutic strategies for uremic pruritus, with an emphasis on emerging treatments and evolving mechanistic insights. **Methods:** A PubMed search was conducted for original clinical studies published between 1 January 2015, and 31 October 2025, evaluating treatments for uremic pruritus in adult hemodialysis patients. Eligible study designs included randomized controlled trials and observational interventional studies. Non-English articles, pediatric studies, peritoneal dialysis studies, reviews, case reports, and studies of mixed-etiology pruritus were excluded. Earlier literature was reviewed to contextualize epidemiology and pathophysiology. **Results:** The review identifies multiple interacting mechanisms—including uremic toxins, immune dysregulation, mineral abnormalities, xerosis, neuropathic changes, and dysregulated opioid signaling—contributing to itch generation. Topical therapies, especially emollients and humectants, consistently improved symptoms with excellent safety profiles. Optimization of dialysis adequacy and membrane selection showed benefit in selected patients. Among systemic therapies, gabapentinoids demonstrated the most robust efficacy but required cautious dosing. Sertraline, nalbuphine, and difelikefalin showed significant antipruritic effects in controlled trials. Emerging therapies, including AST-120, omega-3 fatty acids, and the biologic dupilumab, demonstrated promising but preliminary results. **Conclusions:** Management of uremic pruritus requires a multifaceted, individualized approach integrating skin-directed therapies, dialysis optimization, and targeted systemic treatments. Ongoing research is needed to identify reliable biomarkers and to develop safer, more effective, mechanism-based therapies.

## 1. Introduction

Uremic pruritus is an itching sensation experienced by patients with chronic kidney disease (CKD), the etiology of which is multifactorial and not completely understood. The reported prevalence among patients on intermittent hemodialysis (HD) varies widely, with studies reporting rates ranging from 25% to 73% and meta-analyses reporting prevalence of 52% and 55% [1,2,3,4]. This variability appears to arise primarily from inconsistent definitions and assessment tools (e.g., any itch versus moderate-to-severe itch in questionnaires), as well as methodological differences across studies (point prevalence versus lifetime prevalence). Additionally, geographic and population-related factors, dialysis adequacy, residual kidney function, and associated metabolic disturbances all contribute to the observed differences. It is often overlooked by healthcare professionals and underreported by patients [5,6]. Nevertheless, uremic pruritus significantly impairs quality of life through sleep disruption, chronic fatigue, and limitations in daily functioning, and has been associated with psychological consequences including depression, anxiety, and reduced social well-being [7,8,9,10]. Beyond these effects, severe pruritus has been linked to skin damage, secondary infections, and increased cardiovascular mortality [11,12]. Population-based data further indicate increased risks of infection-related hospitalizations, catheter-related infections, and major cardiovascular and cerebrovascular events among patients reporting pruritus [13].

Although the understanding of its pathophysiology has evolved, effective management of uremic pruritus remains challenging. In this narrative review, we summarize and critically discuss recent clinical evidence on the pathophysiology and management of uremic pruritus in patients receiving HD, with the aim of providing a clinically relevant overview to support evidence-based decision-making.

## 2. Methods

This article was designed as a narrative review aimed at providing a focused, clinically oriented synthesis of recent evidence on the management of uremic pruritus in adult patients undergoing maintenance hemodialysis.

A focused literature search was conducted in the PubMed database to identify original clinical studies evaluating therapeutic interventions for uremic pruritus. The search covered publications from 1 January 2015, to 31 October 2025, in order to reflect contemporary treatment strategies, trial designs, and evolving mechanistic insights. The following search terms were used in various combinations: “uremic pruritus,” “chronic kidney disease–associated pruritus,” “hemodialysis,” “itch,” “chronic renal failure,” “treatment,” “gabapentin,” “pregabalin,” “difelikefalin,” “nalbuphine,” “topical therapy,” “emollient,” “xerosis,” and “dialysis adequacy.” Boolean operators (AND, OR) were applied to refine the search. In addition, reference lists of relevant articles and selected review papers were examined to identify additional pertinent studies.

Eligible publications included randomized controlled trials, prospective or retrospective cohort studies, and other interventional clinical studies assessing treatments for uremic pruritus in adult hemodialysis patients. Studies were excluded if they were non-English publications, involved pediatric populations or patients receiving peritoneal dialysis, addressed pruritus of non-uremic or mixed etiology, or were case reports, editorials, conference abstracts, systematic reviews, or meta-analyses. Pediatric populations and peritoneal dialysis patients were excluded to maintain clinical homogeneity, as the epidemiology, pathophysiology, and management of pruritus differ across age groups and dialysis modalities. Case reports were generally excluded to focus the narrative on interventional evidence with broader clinical applicability; however, in the context of emerging therapies for which larger clinical trials are lacking, selected case reports and small case series were considered to illustrate early clinical experience and potential therapeutic signals. Systematic reviews and meta-analyses were excluded to avoid duplication of existing syntheses and to allow direct discussion of original clinical studies.

Article selection and data extraction were performed by two authors, with disagreements resolved through discussion and consensus. Extracted information included study design, sample size, intervention characteristics, treatment duration, outcome measures, principal findings, and reported adverse effects.

As this was a narrative review, no formal risk-of-bias assessment, deduplication process, or quantitative synthesis was performed. Owing to heterogeneity in study designs, interventions, and outcome measures, findings were summarized descriptively and discussed narratively. Earlier literature published before 2015 was reviewed selectively to provide essential background on epidemiology, pathophysiology, and clinical characteristics of uremic pruritus, but was not subject to structured eligibility criteria.

For consistency, the term ‘uremic pruritus’ is used throughout this review to describe pruritus occurring in patients receiving maintenance hemodialysis, unless otherwise specified.

OpenAI’s ChatGPT (OpenAI, https://chat.openai.com, accessed on 26 December 2025) was used to assist in the creation of the conceptual figure layout. The figure was subsequently reviewed, refined, and approved by the authors, who take full responsibility for its accuracy and clarity.

## 3. Discussion

The discussion integrates current understanding of the multifactorial pathophysiology of uremic pruritus with its clinical implications and therapeutic challenges in patients receiving maintenance hemodialysis.

### 3.1. Pathophysiology

**Figure 1:**
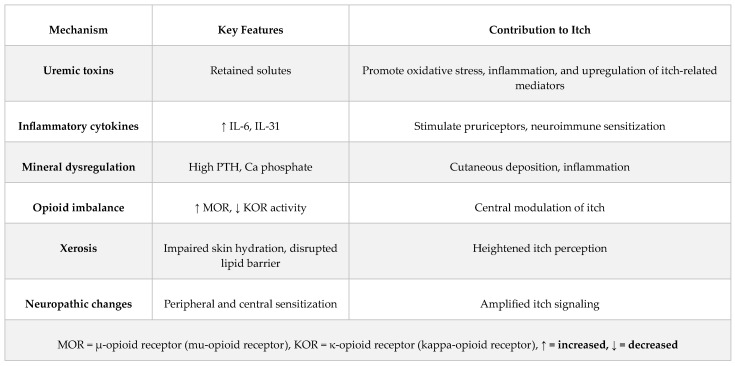
Proposed mechanisms involved in the pathophysiology of uremic pruritus and their contributions to itch generation.

**Figure 2:**
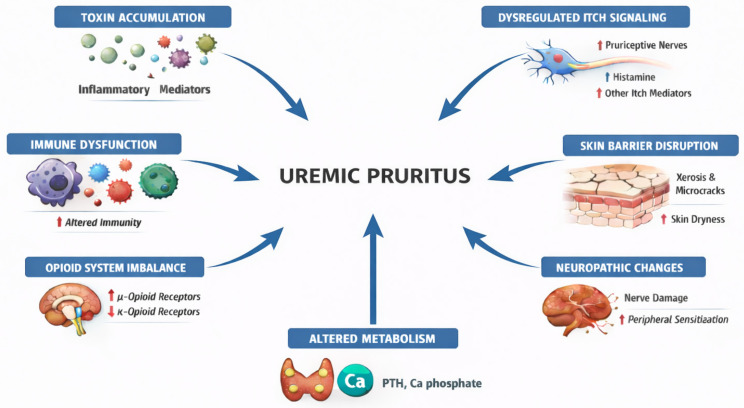
Proposed mechanisms underlying uremic pruritus. The blue arrows indicate directional relationships and contributory pathways toward uremic pruritus, while the red up/down arrows denote increased or decreased levels or activity of the indicated mediators or receptors.

The pathogenesis of uremic pruritus is multifactorial and not fully understood. In addition to the accumulation of uremic toxins, one of the most extensively studied mechanisms, several other interrelated mechanisms have been identified and are to be discussed in this section (Figure 1 and Figure 2).

Beta-2 microglobulin (B2M) is a low-molecular-weight protein, forming a part of the Major Histocompatibility Complex (MHC) Class I molecule present on all nucleated cells. Kidneys play a critical role in their degradation in healthy individuals. Consequently, B2M levels rise in patients on chronic hemodialysis. Based on animal studies, B2M appears to exert pruritogenic effects [14]. Protein-bound uremic toxins like indoxyl sulfate (IS) and p-cresyl sulfate (PCS) are also frequently mentioned in the pathogenesis of uremic pruritus. These toxins are generated by gut microbial metabolism of tryptophan, tyrosine, and phenylalanine, then sulfated in the liver/intestine, and in CKD, they accumulate due to reduced renal excretion [15]. In addition, they are insufficiently removed by hemodialysis filters because they are tightly bound to albumin [15]. IS and PCS have been shown in experimental settings to promote inflammation, oxidative stress, and upregulation of itch-related mediators (e.g., expression of protease-activated receptor-2 in keratinocytes), and correlate in some studies with pruritus severity [16,17,18,19,20,21]. They are also postulated to affect immune cell behavior by activating pattern recognition receptors (e.g., Toll-like receptors (TLRs) expressed on keratinocytes), inducing oxidative stress, and disrupting gut barrier integrity, thereby creating a feedback loop that sustains systemic and cutaneous inflammation [22]. Moreover, in a recent clinical study, administration of the oral adsorbent AST-120 for four weeks resulted in a significant reduction in pruritus severity, as will be discussed later in the text [23]. Nevertheless, the influence of uremic toxins and their clearance by the dialysis filter on uremic pruritus is debated and will be further discussed in the treatment section [24]. In the setting of extreme, untreated uremia, excessive accumulation of urea may give rise to uremic frost, a rare cutaneous manifestation characterized by crystallization of urea on the skin surface and often accompanied by intense pruritus [25]. With the widespread availability of HD, this finding has become exceedingly uncommon and is now primarily of historical relevance rather than a feature of contemporary dialysis-associated uremic pruritus.

Immune dysregulation is also recognized as one of the factors influencing the development of uremic pruritus. Chronic kidney disease creates a pro-inflammatory milieu with elevated systemic markers such as C-reactive protein (CRP), interleukins (IL) IL-6, IL-2, and IL-31, along with perturbations in T cell subsets and their activation status [26,27]. In particular, IL-31, a cytokine produced by activated T cells, has been consistently found at higher concentrations in pruritic CKD patients compared to non-pruritic controls, and is hypothesized to contribute to itch via IL-31 receptor signaling on keratinocytes and sensory neurons [28,29,30]. Moreover, interactions between IL-31, mast cells, and keratinocytes may sustain a local inflammatory loop that amplifies itch perception [31]. Taken together, immune dysregulation in CKD is not only a marker of generalized inflammation but likely acts as a driver of cutaneous neuroimmune sensitization, offering potential therapeutic targets such as IL-31 antagonists and immunomodulators.

Mineral dysregulation is another contributor to the pathogenesis of uremic pruritus. Disruptions in calcium–phosphate homeostasis and secondary/tertiary hyperparathyroidism result in elevated serum phosphate levels, increased calcium–phosphate product, and elevated parathyroid hormone (PTH) levels, all of which have been correlated with increased pruritus severity [32,33,34]. These abnormalities promote ectopic calcium phosphate deposition in the skin and vasculature. Calcium phosphate is directly pruritogenic in animal models, eliciting itch responses upon intradermal injection through the induction of IL-6 signaling, which amplifies downstream pathways involved in sensory neuron activation [35]. In contrast, parathyroid hormone (PTH) itself has not been shown to exert direct pruritogenic effects when administered intradermally [36]. Although earlier hypotheses proposed that CKD-associated pruritus was closely linked to abnormalities in phosphorus, calcium, and PTH, more recent clinical studies have failed to confirm these associations [37,38].

Alteration of the opioid system has been implicated in the development of pruritus in CKD patients. There are 3 subtypes of receptors: μ (mu; MOR), κ (kappa; KOR), and δ (delta; DOR) [39]. They are expressed not only in the central nervous system but also in peripheral nerves, keratinocytes, mast cells, and other skin-resident cells [40,41]. Under physiological conditions, a balance exists between these systems: MOR activation (e.g., by β-endorphin or exogenous opioids) inhibits pain pathways but can stimulate itching sensation [42,43,44]. Conversely, KOR activation (e.g., by endogenous dynorphins) counteracts itch by inhibiting MOR-mediated excitation and suppressing itch-specific spinal interneurons [39,42,45,46]. Immunohistochemical studies on the skin of hemodialysis patients have shown reduced expression of KOR in pruritic versus non-pruritic individuals, and a negative correlation between KOR density and itch intensity [47]. Furthermore, clinical trials have shown that KOR agonists (and mixed MOR antagonists/KOR agonists) reduce itch severity in this population, as will be discussed later in this review [48].

Xerosis, or abnormally dry skin, is one of the most prevalent dermatologic manifestations in patients with CKD and appears to be closely associated with uremic pruritus. The pathogenesis of xerosis in uremic patients is multifactorial. Impaired sweat and sebaceous gland function, reduced skin surface lipids, and alterations in epidermal barrier integrity contribute to transepidermal water loss and skin dryness [49,50,51]. Xerosis may exacerbate pruritus by enhancing cutaneous nerve ending exposure and reducing the itch threshold [52].

Uremic neuropathy, which frequently affects patients undergoing chronic hemodialysis, is thought to result from the accumulation of uremic toxins, oxidative stress, and metabolic derangements that damage peripheral small nerve fibers [53,54]. This peripheral nerve injury lowers the excitatory threshold of pruriceptive neurons, promoting aberrant neuronal firing and peripheral sensitization [55]. Moreover, central sensitization occurs in the spinal cord, where dorsal horn and supraspinal neurons are affected by increased excitatory signaling or altered inhibitory signaling of interneurons [55]. Clinical support for the neuropathic hypothesis includes the higher prevalence of neuropathy among HD patients with pruritus compared to those without [56], and the efficacy of neuropathic-modulating agents (e.g., gabapentinoids) in alleviating itch in dialysis populations [57].

### 3.2. Assessment and Outcome Measures

Since itching is highly prevalent among patients with CKD, it is often presumed to be uremic in origin. Nevertheless, a thorough evaluation is necessary to exclude other etiologies, including skin conditions, liver disease, neuropathic and psychogenic disorders, endocrine abnormalities, as well as drug-induced pruritus [58,59]. Certain medications, such as diuretics, beta blockers, angiotensin-converting enzyme (ACE) inhibitors, clonidine, and allopurinol, may induce itch through the activation of μ-opioid receptors [60]. Another important consideration is infectious causes, such as scabies, which, if not promptly identified and treated, can quickly result in an epidemic in the dialysis unit [61,62]. Considering the broad etiology of pruritus, failure to consider scabies in the differential diagnosis may lead to cumbersome additional testing, unnecessary costs, and delayed recognition of disease spread, which in turn may result in ward closure and further technical and organizational issues [63,64].

Initial evaluation of itch should include a complete blood count with differential, fasting glucose, liver and kidney function tests, and thyroid function tests to exclude other systemic etiologies. A detailed clinical history and physical examination may further guide the need for additional diagnostic testing [58].

Skin examination often reveals dry skin or xerosis, present in 50–85% of patients with uremic pruritus, characterized by cracking and scaling upon closer inspection [65]. Secondary skin changes arise from repetitive scratching and include efflorescences such as excoriations, erosions, and crusts, as well as conditions like lichen simplex chronicus and prurigo nodularis. It is important to differentiate these from other primary dermatological conditions, such as scabies, atopic dermatitis, contact dermatitis, lichen planus, psoriasis vulgaris, autoimmune blistering diseases, drug eruptions, chronic urticaria, etc. Referral to a dermatologist may assist in establishing the correct diagnosis of pruritus [31,58].

Uremic pruritus may present as either localized or generalized itching, with considerable variability in its distribution and timing. Localized pruritus commonly affects the head, back, and arms in a symmetric fashion, but it is often discontinuous, and any body region can be affected. The relationship between pruritus and dialysis varies among patients, with some reporting more severe itching before dialysis sessions and others after treatment [65,66]. Many patients experience symptoms daily. Itch intensity often peaks at night, leading to disrupted sleep and impaired quality of life [65].

Studies have shown inconsistent findings regarding the association of pruritus with age [3,6] and gender [67,68]. Comorbid conditions such as cardiovascular disease, diabetes mellitus, neurologic disorders, depression, liver disease, and lung disease have been linked to more pronounced symptoms [68].

Since uremic pruritus often occurs without evident skin changes, its recognition may be delayed, as well as its severity [6]. Clinicians may overlook or fail to inquire about pruritus due to other health concerns, limited knowledge about uremic pruritus, and a lack of effective treatment options. Conversely, patients frequently underreport pruritus, which may be influenced by a lack of awareness of its link to chronic kidney disease, adaptation to the symptom, communication barriers, or limited time during their appointment with the clinician [69].

The impact of pruritus can be routinely assessed using patient-reported outcome measures (PROMs). These standardized tools facilitate the identification of uremic pruritus and its effects on patients’ daily lives. By employing such questionnaires, health care providers can monitor changes in symptom severity over time, making it easier to evaluate the effectiveness of treatments. Tools may be unidimensional (focusing solely on itch intensity) or multidimensional (capturing both the severity of pruritus and its impact on health-related quality of life [HR-QoL]) [70,71].

The Visual Analog Scale (VAS) is a unidimensional tool that uses a graphic scale on which patients indicate a point that describes the severity of their symptoms over the preceding 24 h (0 represents no itching, and 10 represents the worst imaginable itching) [71].

Worst Itch Numerical Rating Scale (WI-NRS) is similar to VAS, but patients are asked to select a number that best represents itching severity. Pruritus can then be classified as mild (1–3), moderate (4–6), severe (7–8), and very severe (≥9). This tool has demonstrated efficacy in randomized clinical trials, is simple to use, and its scores align closely with those of multidimensional tools [72]. Furthermore, question 20 of the KDQOL-36 (Kidney Disease Quality of Life—36 Item Short Form Survey) asks respondents to indicate the extent to which they have been bothered by dry skin over the preceding four weeks [73].

Multidimensional aspects of pruritus may be assessed using the 5-Dimensional Itch Scale (5-D Itch), which evaluates five domains: degree, duration, direction (change over time), disability (impact on daily activities), and distribution (body areas affected). This instrument provides a broader assessment of pruritus burden and allows standardized comparisons [74].

The Skindex-10 and ItchyQoL are dermatology-specific quality-of-life instruments designed to assess the psychosocial and functional consequences of pruritus [70].

### 3.3. Treatment Options

Owing to the limited understanding of the pathogenesis of uremic pruritus and the absence of consensus guidelines, effective management of uremic pruritus remains challenging.

#### 3.3.1. Topical and General Measures

Since xerosis is commonly observed in affected patients, regular skin hydration is recommended irrespective of disease severity [70]. The recently published 2025 European guideline on the management of pruritus provides updated recommendations for implementing general pruritus-relieving measures alongside specific treatments [75]. Patients are advised to wear soft, breathable fabrics, such as cotton, and to apply moisturizers frequently to prevent skin dryness. Wearing layered outfits and maintaining a cool room temperature at night can help avoid overheating and sweating. Fingernails should be kept short to minimize skin damage from scratching. Dietary triggers, such as spicy foods, very hot beverages, and alcohol, are often associated with worsening of symptoms and should be avoided when possible. When bathing, patients are advised to use lukewarm water in combination with gentle, fragrance-free soaps, bath oils, or moisturizing cleansers. The skin should be patted dry rather than rubbed, followed by immediate application of a moisturizer. Patients should limit their exposure to drying or irritating factors, such as frequent showers, saunas, and compresses containing alcohol or other irritants. For symptomatic relief, cooling wet wraps may be beneficial [70,75]. In addition, patient education on coping strategies for the itch–scratch cycle is vital for overall management [76].

The use of moisturizers or emollients is fundamental for restoring the skin barrier and represents the initial step in the management of uremic pruritus. These topical preparations are commonly available in the form of ointments, creams or lotions. When applied regularly, they can substantially reduce the severity of pruritus and, in some patients, even completely abolish symptoms [77]. Skin hydration, barrier integrity and transepidermal water loss are important factors influencing itch sensation in hemodialysis patients [78].

Both moisturizers and emollients consist of lipids that create an occlusive layer on the skin surface, thereby reducing the transepidermal water loss. Moisturizers additionally contain humectants, i.e., small molecules that attract and retain water into the stratum corneum, such as urea, glycerin, dexpanthenol, hyaluronic acid, and propylene glycol. Furthermore, certain lipid components and humectants (such as N-palmitoylethanolamine, polidocanol and menthol) possess mild analgesic and antipruritic properties, contributing directly to itch relief [75].

A recent multicenter, randomized, double-blind study was conducted among 235 dialysis patients with moderate-to-severe uremic xerosis, investigating the efficacy of an emollient containing 15% glycerin and 10% paraffin compared with its vehicle. Patients who applied the emollient containing glycerin and paraffin achieved greater response rates (60.2% vs. 41.0%; *p* = 0.0041) and greater reductions in scaling, with effects persisting after treatment cessation. Although the tested emollient showed greater improvement of xerosis, both groups applied oil-in-water emulsions, which likely contributed to the significant reductions in pruritus observed in both groups [79]. Clinical trials have also demonstrated the benefits of other previously mentioned humectants and lipid components (Table 1) [65,80]. Irritants, fragrances, and preservatives should be avoided, as these substances may exacerbate itch and, in some cases, induce contact allergies [75].

Emollients and moisturizers address the underlying xerosis in patients with uremic xerosis and should be encouraged at least once daily, due to their effectiveness, ease of use, and excellent safety profile [80].

Capsaicin, a phytochemical derived from the plant genus *Capsicum*, has been used to alleviate itch by depleting substance P, a neuropeptide that transmits pain and itch signals from sensory nerve terminals [65,81]. Interest in the use of topical capsaicin for uremic pruritus peaked in the 1990s, when most clinical trials investigating its efficacy were conducted. Across these studies, topical capsaicin demonstrated notable improvements, with pruritus scores decreasing by 57% to 84% [81]. However, subsequent analyses found only limited overall efficacy [65]. A significant methodological obstacle in study designs is the burning sensation caused by topical capsaicin, which cannot be replicated by placebo creams, compromising blinding in clinical trials. Furthermore, patients using topical capsaicin report a relatively high rate of adverse events, namely burning/tingling sensations, moderate skin burning, and erythema [81]. Consequently, prescribing topical capsaicin requires consideration of patients’ ability to tolerate these potential adverse effects.

**Table 1:** Selected clinical trials on the topical treatment of CKD-associated pruritus.

Author, Year [Reference Number]	Study Design	Nr of Participants	Treatment Duration	Treatment Method	Method of Assessment	Results
Szepietowski (2024) [79]	Phase 3, randomized, double-blind, vehicle-controlled study	235	Period I: 28 days 1× daily (V0034CR or vehicle) Period II: ≤21 days treatment-free Period III: ≥84 days open-label, V0034CR for all patients	Topical application of V0034CR (emollient containing glycerol 15% + paraffin 10%) vs. vehicle, 1× daily	VAS, El Gammal score (0–4), DLQI	Treatment response (Period I): 60.2% V0034CR vs. 41.0% vehicle (*p* = 0.0041) Partial maintenance in Period II; full recovery and maintenance during Period III (low recurrence, 2.1%) AEs were irritation or erythema (2.1%), exacerbated pruritus (1.3%), and vesicles at the application site (0.9%). Comparable improvement in pruritus between V0034CR and vehicle, suggesting the base emulsion is largely responsible
Singh vs. et al. (2021) [82]	Experimental pre-test and post-test control group design	120	10 days	Baby oil containing mineral oil + Vitamin E, massaging for 15 min 1× daily	NRS	Experimental group: Significant reduction in itching—mean pruritus reduction: 23.7% (difference 2.37 points). After treatment: 20% had no itch; none had severe itch Control group—mean pruritus reduction: 1.3% (difference 0.13 points)
Nevols J et al. (2023) [83]	Phase IV randomized, double-blind, controlled, parallel-group trial	58	4 weeks	Balneum Plus cream (3% lauromacrogols + 5% urea) vs. standard emollient 2× daily	VAS	No significant difference in itch score reduction between groups (*p* = 0.64). Balneum group median baseline VAS 6.5—decreased to 2.6 Emollient group median baseline VAS 6.3—decreased to 2.0 No significant difference in AE
Yahya YF et al. (2018) [84]	Randomized, double-blind, placebo-controlled clinical trial	65	4 weeks	20% urea cream in a base of NaPCA, sodium lactate, and vegetable oils vs. vehicle with the same base without urea	VAS, skin hydration (corneometer)	20% Urea group improved significantly compared to placebo: VAS decreased from 5.58 ± 1.25 to 2.78 ± 1.07 vs. placebo 5.64 ± 1.37 to 4.70 ± 1.83 (*p* < 0.001) Skin hydration improved significantly in urea group compared to placebo (*p* < 0.001)
Muliani R et al. (2021) [85]	Quasi-experimental, two-group pre/post-test design	72	14 days	Virgin Coconut Oil group (VCO) Olive Oil (OO) group Applied 2×/day	Duo’s grade of pruritus	Both oils produced reductions in pruritus grade scores. VCO group showed a greater reduction compared to olive oil (*p* = 0.008), indicating VCO was more effective.
Aquino TMO et al. (2020) [86]	Randomized, double-blind, vehicle-controlled clinical trial	30	14 days	Topical 6% gabapentin cream vs. vehicle, 1× daily	VAS, MCPS-VAS	MCPS-VAS of the two groups were not significantly different (*p* = 0.8) after 1 week After 2 weeks, MCPS-VAS of the topical gabapentin group was greater (−4.6) (*p* = 0.01) compared to control (−2.6) No side effects reported
Anumas S et al. (2024) [87]	Randomized, double-blind, placebo-controlled clinical trial	60	4 weeks	Cannabis-containing cream (5% Cannabis sativa oil) vs. placebo cream, 2× daily	WI-NRS, Skindex-10	WI-NRS at week 2: no significant difference between groups At week 4, the cannabis group significantly improved vs. placebo (adjusted mean difference −1.1, *p* = 0.02) Skindex-10: Lower (better) scores in the cannabis group at weeks 2 and 4, but no significant difference after baseline adjustment
Widyastuti et al. (2021) [88]	Randomized, double-blind, placebo-controlled clinical trial	65	4 weeks	Topical calcipotriol 0.005% ointment vs. placebo ointment, 2× daily to lower legs	VAS, ODSS, SHL, LSS	Both groups improved, more significantly the calcipotriol group (*p* < 0.01) Calcipotriol group vs. placebo (ODSS; 83.9% vs. 29%, VAS; 74.2% vs. 19.4%, skin hydration; 96.8% vs. 19.4%, lipid layer; 80.6% vs. 22.6%)
Khorsand A et al. (2019) [89]	Randomized controlled trial	52	6 sessions/2 weeks	Massage with 5 mL violet oil for 7 min	Pruritus severity (questionnaire-based)	Both groups improved significantly; massage with violet oil was more effective than massage alone (*p* < 0.05)
Elsaie LT et al. (2016) [90]	Randomized controlled trial	50	2 weeks	Topical 5% peppermint oil vs. topical petrolatum (placebo), 2× daily	5-D itch scale	Peppermint group: significant improvement in total 5-D IS score (15.18 → 7.94; *p* < 0.05) and all domains except “work” or “work + housework” for some Placebo group: no significant improvement in total 5-D IS score (14.54 → 13.47) or most domains 4 patients reported mild burning sensation in flexures; otherwise, no AEs

VAS—Visual Analog Scale; DLQI—The Dermatology Life Quality Index; NRS—Numerical Rating Scale; NaPCA—sodium pidolat sodium lactate; AE—Adverse Events; MCPS-VAS—mean change in pruritus scores using the VAS; WI-NRS—Worst Itch Numerical Rating Scale; ODSS—Overall dry skin score; SHL—skin hydration levels; LSS—Lipid skin surfaces level; VCO—Virgin Coconut Oil; OO—Olive Oil, Nr—number.

#### 3.3.2. Dialysis-Related Interventions

Despite a declining incidence of pruritus in hemodialysis patients over time, it remains a relevant issue in a significant proportion of the hemodialysis population [6,91]. A considerable number of patients undergoing dialysis do not report pruritic symptoms, highlighting that increased awareness and timely diagnosis represent initial steps toward improvement [69]. Because of its substantial effect on quality of life and patient management, pruritus can be used as a key performance indicator or quality indicator in dialysis units [92].

Enhancing the clearance of uremic toxins through adjustments in dialysis dose or dialyzer membrane characteristics has been postulated to play a role in the treatment of uremic pruritus. In pre-dialysis patients, pruritus severity worsens with each 5 mL/min/m^2^ decrease in estimated glomerular filtration rate and improves following kidney transplantation [93,94]. Accordingly, improved molecular clearance through dialysis would be expected to alleviate pruritus; however, available studies have shown inconsistent results. A likely explanation is that the specific pruritogenic substances that can be removed by dialysis have not been clearly identified. Regardless, the presence of pruritus should prompt clinicians to reevaluate the dialysis protocol with particular attention to dialysis adequacy. These recommendations are supported by evidence demonstrating that inadequate dialysis is associated with greater pruritus severity, and improving dialysis adequacy to a Kt/V > 1.2 has been beneficial in a subset of patients [95]. Considering that suboptimal dialysis adequacy is associated with poorer clinical outcomes and lower quality of life, measures to improve dialysis adequacy to a target Kt/V of approximately 1.4 are recommended in patients with a Kt/V < 1.2, irrespective of the presence of pruritus [96].

Interestingly, in a large multinational multicenter study, differences in Kt/V had no impact on pruritus prevalence, despite an earlier phase of the same study demonstrating that pruritus was more frequent in patients with a Kt/V < 1.2 than in those with a Kt/V > 1.5 [6,97]. In contrast, a large prospective study by Ko et al. showed that lower Kt/V values and an initial Kt/V < 1.5 were independent predictors of pruritus severity. In the same study, the use of a high-flux dialyzer membrane was associated with a lower likelihood of severe pruritus [98]. This effect may be related to improved B2M clearance, as B2M has been independently associated with pruritus, and enhanced clearance has been linked to symptom improvement [34]. High-flux filters have similarly been associated with improved pruritus compared with low-flux filters in other studies [99]. Additionally, the use of polymethylmethacrylate (PMMA) membranes has been associated with pruritus improvement, possibly due to enhanced B2M removal despite no changes in Kt/V [100,101]. PMMA membranes possess a uniform structural composition and a high adsorptive capacity [102]. In a prospective interventional study involving 20 hemodialysis patients, Takahashi et al. demonstrated that switching to a PMMA membrane hemodiafilter resulted in a significant reduction in pruritus by Week 2, with sustained improvement through Week 12 [103]. Throughout the study, investigators also monitored concomitant antipruritic therapies, including moisturizers, topical corticosteroids, other topical agents, oral antihistamines, nalfurafine, and γ-aminobutyric acid (GABA) receptor agonists, to ensure that observed changes in pruritus severity were not attributable to alterations in background treatment. Another potential etiological factor influencing pruritus severity is patient reactivity to the dialyzer membrane. In some cases, switching to a dialyzer composed of more biocompatible materials, such as cellulose triacetate or vitamin E-coated membranes, may be beneficial [104]. Depending on the availability of dialyzer membranes across clinical settings, a trial of filters made from alternative materials is warranted when pruritus is suspected to result from an allergic or hypersensitivity reaction to the dialyzer. Dialyzers made from cellulose derivatives can be safely used in patients with suspected or confirmed dialyzer hypersensitivity, as they have not been associated with hypersensitivity reactions in a large multicenter Spanish report of 1561 dialysis patients by Esteras R et al. [105]. In patients exhibiting reactions to polysulfone or polyethersulfone dialyzers, alternatives such as modified cellulose, PMMA, or polyacrylonitrile dialyzers may be used [106].

In patients already receiving high-flux hemodialysis, the use of medium cut-off filters may be considered to further enhance middle-molecule clearance. In a Korean randomized trial, medium cut-off filters were associated with superior PROMs compared with high-flux filters [107]. Increasing blood pump flow represents another strategy to improve solute clearance, as demonstrated by an Iranian study reporting improvement in pruritus severity following higher blood flow rates during dialysis [108].

Another approach to increasing targeted molecular clearance is the addition of hemadsorption to the dialysis regimen. The use of hemadsorption in combination with hemodialysis has been shown to enhance B2M clearance and to improve pruritus severity irrespective of dialyzer type (low- vs. high-flux) [109]. However, hemoperfusion combined with high-flux hemodialysis appears to be more effective in reducing symptoms than hemoperfusion combined with low-flux hemodialysis.

#### 3.3.3. Systemic Pharmacotherapy

A wide range of pharmacologic agents has been investigated for the treatment of uremic pruritus among HD patients, reflecting the condition’s complex underlying mechanisms (Table 2). Anticonvulsants such as gabapentin and pregabalin are among the most frequently used, as they reduce neuronal excitability and help modulate abnormal sensory signaling pathways [110]. However, since these drugs are primarily excreted by the kidneys, accumulation in patients with renal impairment can lead to neurotoxicity, which represents the main safety concern in this population [111]. Antidepressants like doxepin and sertraline may relieve pruritus by influencing histamine and serotonin activity, whereas antihistamines such as dexchlorpheniramine and ketotifen act directly on histamine-related pathways. Other agents include serotonin (5-HT_3_) receptor antagonists like ondansetron, which block serotonin-mediated itch transmission [112], and opioid receptor modulators, such as nalbuphine and difelikefalin, which restore balance to central and peripheral opioid systems. The following studies summarize clinical evidence comparing the efficacy and safety of these different drug groups in patients with dialysis-associated uremic pruritus.

A randomized, double-blind, placebo-controlled trial evaluated the efficacy and safety of two fixed-dose gabapentin regimens (100 mg and 300 mg after dialysis sessions) versus placebo in hemodialysis patients with severe uremic pruritus [116]. 

Among 21 HD patients, the 100 mg gabapentin group showed a significant reduction in itch severity and fewer nighttime awakenings due to itching, whereas the 300 mg dose resulted in severe sedation in two patients. The study concluded that low-dose gabapentin (100 mg post-dialysis) is effective and well-tolerated, supporting a cautious, stepwise dosing strategy to balance efficacy and neurotoxicity risk. Positive effects of pregabalin were confirmed in a randomized placebo-controlled study involving 54 patients by Nofal E et al. [123], which reported a markedly higher response rate in the gabapentin group compared with placebo, with most patients responding to a low dose of 100 mg thrice weekly. Khan et al. conducted a comparative study of pregabalin (75 mg/day) and gabapentin (300 mg/day) in 90 HD patients with uremic pruritus [57]. Over a six-week treatment period, both drugs significantly reduced pruritus severity, with pregabalin producing a greater reduction in itch scores than gabapentin. However, adverse effects such as sedation, nausea, and blurred vision were more prevalent in patients treated with pregabalin. Notably, this study did not include a placebo control group.

Furthermore, two studies compared the antipruritic effects of doxepin and gabapentin/pregabalin. In a crossover randomized study involving 14 HD patients, Haber R et al. found that both gabapentin and doxepin effectively reduced uremic pruritus, with gabapentin demonstrating greater efficacy [113]. However, the study was limited by a small sample size and the absence of placebo control. Another study involving 72 HD patients found that pregabalin’s antipruritic effects were superior to those of doxepin over a four-week treatment period, with noticeable improvement observed one week after initiation. Both drugs caused somnolence at similar rates [117]. Gobo-Oliveira M et al. compared the efficacy and safety of gabapentin and dexchlorpheniramine in 60 patients with persistent pruritus [114]. After an initial 15-day trial with cold cream, patients were randomized to receive either gabapentin (300 mg three times weekly) or dexchlorpheniramine (6 mg twice daily) for 21 days. Both treatments significantly reduced itch intensity and improved quality of life, with no significant difference between the two groups; side effects such as mild drowsiness were reported in about one-third of participants. Another study compared the effectiveness of pregabalin (50 mg three times daily) to ketotifen (1 mg twice daily) among 30 hemodialysis patients. It found that pregabalin significantly reduced pruritus severity and improved quality of life compared with ketotifen [120]. The study was conducted over 4 weeks on HD patients who failed to respond to dermal emulsion treatment for at least 3 months. Side effects were mild and did not differ significantly different between groups. Yue et al. compared the efficacy of pregabalin and ondansetron to placebo in 188 dialysis patients with uremic pruritus over 12 weeks [115]. Patients were randomly assigned to receive pregabalin, ondansetron, or placebo, with pruritus severity, sleep quality, and quality of life assessed throughout the study period. Five patients discontinued pregabalin treatment owing to adverse effects, including somnolence, severe dizziness, and loss of balance. Two patients in the ondansetron group withdrew due to nausea and vomiting, and an additional two participants discontinued following renal transplantation. Notably, complete resolution of pruritus was observed in the patients who underwent transplantation. The results demonstrated that only pregabalin significantly reduced pruritus severity, whereas ondansetron showed no greater effect than placebo.

A randomized, double-blind, placebo-controlled clinical trial by Elsayed et al. evaluated the efficacy of sertraline among 60 hemodialysis patients [118]. Participants received either sertraline 50 mg twice daily or placebo for eight weeks, with itch severity assessed using the VAS and the 5-D itch scale. The results showed that sertraline significantly reduced pruritus intensity compared with baseline (*p* < 0.001), while the placebo group showed no significant improvement. Another study examined sertraline versus placebo in 50 hemodialysis patients with uremic pruritus [119]. Interestingly, both groups experienced a significant reduction in pruritus severity. However, the decrease was significantly greater in the sertraline group compared to the placebo group. The authors suggested that this effect may be mediated by sertraline’s anti-inflammatory properties, as indicated by correlations with inflammatory markers. In a comparative study of pregabalin (75 mg daily) and sertraline (50 mg daily), pregabalin was significantly more effective in reducing itch severity over a six-week period [122]. Side effects (somnolence) were reported only in the pregabalin group.

Mathur et al. conducted a large clinical trial evaluating the efficacy of nalbuphine extended-release (ER), a μ-opioid antagonist and κ-opioid agonist, in 373 hemodialysis patients with uremic pruritus [48]. Participants were randomized to receive nalbuphine ER 120 mg, 60 mg, or placebo twice daily for eight weeks [48]. The study found that nalbuphine 120 mg significantly reduced itch intensity compared to placebo (mean NRS reduction of 3.5 vs. 2.8; *p* = 0.017), particularly in patients with severe pruritus, who also reported improved sleep quality. Adverse events associated with nalbuphine ER were consistent with those expected from centrally acting opioids. The most common side effects included nausea, vomiting, and somnolence, which occurred primarily during the early dose-titration phase. Approximately 22–26% of patients in the nalbuphine groups discontinued treatment due to these effects, compared with only 6% in the placebo group. However, serious adverse events were infrequent and comparable across treatment arms (6.7–15.4%), with only one case of drug-related vertigo reported. The phase 2 trial evaluated the safety and efficacy of oral difelikefalin (0.25, 0.5, or 1.0 mg once daily for 12 weeks) in 269 patients with CKD stages 3–5, including both non-dialysis-dependent and hemodialysis patients suffering from moderate-to-severe pruritus [121]. The primary endpoint, change in weekly mean WI-NRS score, was significantly improved in the 1.0 mg difelikefalin group versus placebo (*p* = 0.018). The drug was generally well tolerated, with the most common side effects being dizziness, falls, constipation, diarrhea, gastroesophageal reflux disease, fatigue, hyperkalemia, hypertension, and urinary tract infection. No deaths were attributed to the treatment.

#### 3.3.4. Other Systemic Treatments

In a recent clinical study of hemodialysis patients, administration of the oral adsorbent AST-120 for four weeks resulted in a significant reduction in pruritus severity assessed by VAS, which coincided with decreases in serum IS and PCS, as well as inflammatory cytokines such as TNF-α [23]. Mahmudpour et al. conducted a double-blind, randomized trial on 80 HD patients showing that montelukast at a dose of 10 mg daily significantly improved pruritus and lowered inflammatory markers. Follow-up was limited to one month [124]. Baharvand et al. reported similar short-term benefits of melatonin in a small (39 patients) crossover study, but the brief treatment periods and potential carryover effects constrain generalizability [125]. Zanganeh et al. found that both increased blood pump speed and administration of oral activated charcoal reduced itch severity in an unblinded, randomized crossover trial [108].

More recently, Teama et al. performed a randomized, controlled, crossover trial comparing omega-3 fatty acids with gabapentin among 50 HD patients, showing significant reductions in pruritus intensity with both treatments. Omega-3 fatty acid supplementation was connected to reduced prostaglandin E2 (PGE_2_) levels [126]. However, in another crossover trial (40 patients) using 3 g/day of omega-3 versus placebo, the reduction in PGE_2_ was not statistically significant, even though itching improved [127]. An additional randomized controlled study reported a significant reduction in uremic pruritus with omega-3 fatty acid supplementation, while the more recent triple-blind RCT by Rafieipoor et al. did not observe a significant improvement compared with placebo [128,129].

#### 3.3.5. Biologics Targeting Cytokines—Dupilumab

Biologic therapies targeting cytokine-mediated pathways have been investigated in the treatment of uremic pruritus. Dupilumab, a monoclonal antibody that blocks IL-4 receptor α subunit, thereby inhibiting IL-4 and IL-13 signaling, has shown promising benefits in a small case series of 5 patients with chronic kidney disease [130]. In a recently published retrospective observational study of 12 hemodialysis patients, Wang et al. reported a significant reduction in pruritus following dupilumab treatment. The dosing regimen consisted of an initial 600 mg subcutaneous dose, followed by 300 mg every two weeks. Four patients discontinued treatment within the first month because of rapid symptom improvement or financial constraints. The study also observed increased serum IgE levels in some participants and specifically excluded individuals with a history of atopic dermatitis to focus on uremic pruritus. No adverse effects were reported during the study period, supporting the potential safety and tolerability of dupilumab in this patient population [131]. Another retrospective study evaluated dupilumab in patients with CKD, including 12 individuals with atopic dermatitis, six of whom were receiving hemodialysis [132]. Ten patients had uremic pruritus, eight of whom were receiving hemodialysis. All antipruritic medications were discontinued prior to initiation of dupilumab, which was administered using the same dosing regimen and continued for 16 weeks. Both groups of patients had significant reductions in itch scores (PP-NRS, 5-D IS) over time. No serious adverse events were reported; mild conjunctivitis appeared in two patients, and renal function remained stable. Taken together, these findings indicate that current evidence does not allow definitive conclusions regarding the efficacy of dupilumab in isolated uremic pruritus. While limited data suggest a possible benefit in carefully selected patients without atopic dermatitis, the therapeutic effect appears more consistent in patients with chronic kidney disease and coexisting type 2 inflammatory or atopic features. Larger, prospective, controlled trials specifically targeting uremic pruritus are required before dupilumab can be recommended for this indication.

## 4. Conclusions

Uremic pruritus remains a common and clinically significant complication in patients receiving maintenance hemodialysis, with a substantial negative impact on quality of life. Despite increasing insight into its multifactorial pathophysiology, effective management remains challenging, and many patients experience persistent symptoms despite conventional therapies. Recent advances, including targeted neuromodulatory and immunomodulatory treatments, offer promising new options but require further validation in well-designed clinical trials. A deeper understanding of underlying mechanisms and patient-specific factors is essential to improve individualized treatment strategies and clinical outcomes.

## Figures and Tables

**Table 2:** Selected clinical trials on the systemic treatment of CKD-associated pruritus.

Authors, Year [Reference Number]	Study Design	Nr of Participants	Treatment Duration	Drug and Dose	Results	Reported Side Effects
Haber R et al. (2020) [113]	Single-blind crossover randomized trial	14	4 weeks + 4 weeks after a 4-week washout period	Doxepin 10 mg daily or gabapentin 100 mg gabapentin 3× per week. Increase in dose in case of insufficient response	Gabapentin was significantly more effective than Doxepin in reducing the VAS, 5D, and the DLQI	Doxepin: sleepiness, somnolence, drowsiness. Gabapentin: dizziness, nausea
Khan NJ et al. (2022) [57]	Comparative cross-sectional study	90	6 weeks	Pregabalin 25 mg once daily, gabapentin 100 mg once daily	Pregabalin was more efficacious than Gabapentin (*p* = −0.026)	Sedation, nausea, and blurred vision occur more often in pts taking pregabalin
Gobo-Oliveira M et al. (2018) [114]	Randomized, controlled, double-blinded clinical trial	60	21 days	Gabapentin 300 mg 3× per week or dexchlorpheniramine 6 mg 2× per day	Both drugs lead to a significant decrease in pruritus	Sleepiness, fatigue, dizziness, headache, and drowsiness
Yue J et al. (2015) [115]	Prospective, randomized, and double-blind trial	179	12 weeks	Pregabalin 75 mg 2× per week, ondansetron 8 mg daily, or placebo	The severity of pruritus was reduced significantly in the pregabalin group compared with the ondansetron and the placebo groups	Somnolence, dizziness, loss of balance, nausea
Rossi GM at al (2019) [116]	Randomized, double-blind, placebo-controlled trial	21	2 weeks	Gabapentin 100 mg or 300 mg after dialysis sessions, or placebo	Itch reduction was statistically significant in the 100 mg group (*p* = 0.0078)	Lethargy
Foroutan N et al. (2017) [117]	Single-blind multicenter randomized trial	72	4 weeks	Pregabalin 50 mg every other day or doxepin 10 mg daily	Pregabalin was more effective than doxepin in reducing the itch severity	Somnolence in both groups. Pregabalin group: edema, drowsiness, imbalance during walking, and numbness Doxepin: nervousness
Elsayed MM et al. (2023) [118]	Double-blinded, placebo-controlled, multicentric randomized clinical trial	60	8 weeks	Sertraline 50 mg 2× per day or placebo	Significant improvement in pruritus in pts treated with sertraline as compared to placebo	Reported in both groups: xerostomia, dyspepsia, nausea, diarrhea, insomnia. Only reported in sertraline group: headache
Pakfetrat M at al (2018) [119]	Randomized double-blinded placebo controlled clinical trial	50	8 weeks	50 mg sertraline 2× per day or placebo	Pruritus’ intensity decreased significantly more in the sertraline group than in the control group (*p* < 0.001)	None reported
Shamspour N et al. (2022) [120]	Randomized clinical pilot study	30	4 weeks	Pregabalin 50 mg 3× per day, ketotifen 1 mg 2× per day	Pregabalin was more effective than Ketotifen in reducing pruritus	Drowsiness, anxiety, headache, dry mouth, constipation, and diarrhea
Mathur vs. et al. (2017) [48]	Multicenter, randomized, double-blind, placebo-controlled trial	371	8 weeks	Nalbuphine extended-release tablets 120 mg (NAL 120), 60 mg (NAL 60), or placebo	NAL 120 mg significantly reduced itch intensity compared to placebo	Nausea, vomiting, somnolence, vertigo
Yosipovitch G et al. (2023) [121]	Double-blind, randomized, placebo-controlled, dose-finding study	269	12 weeks	Difelikefalin 0.25, 0.5, or 1.0 mg or placebo once per day	Oral difelikefalin significantly reduced itch intensity	Dizziness, fall, constipation, diarrhea, gastroesophageal reflux disease, fatigue, hyperkalemia, hypertension, and UTI
Abbas A et al. (2024) [122]	Randomized controlled trial	48	6 weeks	Pregabalin 75 mg daily or sertraline 50 mg daily	Pregabalin was more effective	Somnolence
Nofal E et al. (2016) [123]	Randomized placebo controlled study	54	One month	Gabapentin 100 mg to 300 mg after each HD session, or placebo	The gabapentin group showed a markedly higher response rate compared to placebo	Dizziness, somnolence, fatigue

VAS—Visual Analog Scale; 5D—5-Domain Itch Scale; DLQI—The Dermatology Life Quality Index; pts—patients; UTI—urinary tract infection, HD—hemodialysis, Nr—number.

## Data Availability

The raw data supporting the conclusions of this article will be made available by the authors on request.
